# Tetraspanin CD81 serves as a functional entry factor for porcine circovirus type 2 infection

**DOI:** 10.1128/jvi.01408-24

**Published:** 2024-12-31

**Authors:** Junshuo Li, Lin Lv, Yanni Gao, Yangyang Sun, Juan Bai, Xianwei Wang, Haifen Sun, Ping Jiang

**Affiliations:** 1 Key Laboratory of Animal Disease Diagnostics and Immunology, Ministry of Agriculture, MOE International Joint Collaborative Research Laboratory for Animal Health & Food Safety, College of Veterinary Medicine, Nanjing Agricultural University 261674 , Nanjing, Jiangsu, China; 2 Jiangsu Co-innovation Center for Prevention and Control of Important Animal Infectious Diseases and Zoonoses, Yangzhou University 38043 , Yangzhou, Jiangsu, China; Michigan State University, East Lansing, Michigan, USA

**Keywords:** porcine circovirus type 2, tetraspanin CD81, virus invasion

## Abstract

Porcine circovirus type 2 (PCV2) is the primary causative agent of porcine circovirus-associated disease, clinically resulting in immunosuppression and co-infections with other pathogens in infected pigs. The mechanism of PCV2 infection remains unclear. In this study, we firstly found that the tetraspanin CD81 in PK-15 cells interacts with PCV2 Cap protein by using virus overlay protein-binding assay combined with mass spectrometry. Knockdown of the CD81 significantly reduces the levels of the viral Cap mRNA and protein, and viral internalization in PK-15 cells. The critical interaction regions locate in the large extracellular loop (LEL) domain of CD81 and the CD loop region (82–91aa) of the Cap protein, and a polyclonal antibody against the CD81 LEL domain significantly inhibits PCV2 infection. The transmembrane proteoglycan Syndecan-1 interacts with both CD81 and PCV2 Cap, and co-operates with CD81 to promote PCV2 infection in PK-15 cells. Furthermore, CD81 facilitates RhoA activation and enhances the viral internalization and replication in PK-15 cells. It was concluded that the tetraspanin CD81 is a key host factor for PCV2 invasion into PK-15 cells, thus providing new insights into PCV2 life cycle and identifying a potential target for antiviral drug development.

**IMPORTANCE:**

Porcine circovirus type 2 (PCV2), a significant economic pathogen in the swine industry, presents persistent challenges in its prevention and treatment. Despite extensive research, the mechanism of PCV2 invading host cells remains unclear. In this study, we found and identified a novel interaction between the tetraspanin CD81 and the viral Cap protein during the PCV2 invading PK-15 cells. The transmembrane proteoglycan Syndecan-1 and RhoA are involved in the infection process through the CD81. Moreover, this is the first time that the role of Syndecan-1 in the PCV2 infection process has been demonstrated. Also, a polyclonal antibody against the CD81 extracellular domain significantly inhibits PCV2 infection in PK-15 cells. It not only enriches our understanding of PCV2 life cycle but also offers new perspectives for the development of antiviral therapeutics against circovirus.

## INTRODUCTION

Porcine circovirus type 2 (PCV2) is a prevalent viral pathogen in swine, belonging to the Circoviridae family and *Circovirus* genus, characterized as a non-enveloped, small, icosahedral-shaped and single-stranded DNA virus (1). PCV2 primarily induces subclinical symptoms and immunosuppression in clinical settings, predisposing to co-infections with other pathogens, such as porcine reproductive and respiratory syndrome virus, *Mycoplasma hyopneumoniae*, bacterial septicemia or pneumonia, and swine influenza virus (2, 3). PCV2 is also found to be associated with several clinical manifestations, such as postweaning multisystemic wasting syndrome, porcine dermatitis and nephropathy syndrome, reproductive failure, and respiratory and enteric diseases, which are collectively known as porcine circovirus-associated disease (4–7). Hence, PCV2 is a significant threat that causes high economic losses to the global swine industry in recent decades.

PCV2 encompasses 11 predicted open reading frames (ORFs), among which ORF2 encodes the Cap protein (8–10). As the sole structural protein of PCV2, Cap protein plays crucial roles in PCV2 virus attachment, internalization, cytoplasmic transport, as well as virus particle assembly and release. PCV2 has an intricate tropism within tissues, exhibiting replication and persistence across various cell types such as myocardial cells, hepatocytes, monocytes (11), lymphoblasts, macrophages (12, 13), as well as epithelial cells, endothelial cells, and fibroblasts (14). PCV2 typically employs a general mechanism for adherence to various host cell surfaces, specifically utilizing heparan sulfate (HS) and chondroitin sulfate B (CS-B) as its attachment receptors (15). However, after attachment, PCV2 adopts distinct pathways, for example, the clathrin-mediated endocytosis (16–18), micropinocytosis (19), or an actin and Rho GTPase-dependent pathway (20), to enter into different cells. These findings suggested that PCV2 displayed a cell-dependent characteristic during virus entry by binding various receptors. Glycosaminoglycan, such as HS and CS, have been reported to be involved in PCV2 infection (15, 21); however, the removal of these substances could not completely inhibit PCV2 infection of target cells (15, 19), indicating that there are other unidentified cell surface molecules involved in PCV2 entry into cells.

CD81, a member of the tetraspanin integral membrane protein family, forms tissue signaling complexes on the cell surface by interacting with other tetraspanins, integrins, MHC (Major histocompatibility complex) class I or II molecules, involved in the adhesion, morphology, activation, proliferation, and differentiation of B and T cells (22–25). CD81 is widely expressed in a variety of cells, including hepatocytes, epithelial cells, fibroblasts, endothelial cells, and most blood cells (26). It comprises four hydrophobic transmembrane helices, connected by a small extracellular loop (SEL), a large extracellular loop (LEL), and an intracellular domain. The human CD81 was identified as a receptor for hepatitis C virus (HCV) as its LEL mediated HCV invasion by interacting with the viral E2 glycoprotein (27, 28). Furthermore, CD81 has been demonstrated to be involved in the infection processes of various viruses, including influenza A virus, human immunodeficiency virus, and herpes simplex virus type 1, among others (29–31). In this study, CD81 was revealed to interact with PCV2 Cap protein and facilitate PCV2 invasion into PK-15 cells, indicating an auxiliary role of CD81 during PCV2 infection.

## RESULTS

### Identification of host cell membrane proteins associated with PCV2 infection into PK-15 cells via virus overlay protein-binding assay (VOPBA)

**Fig 1:**
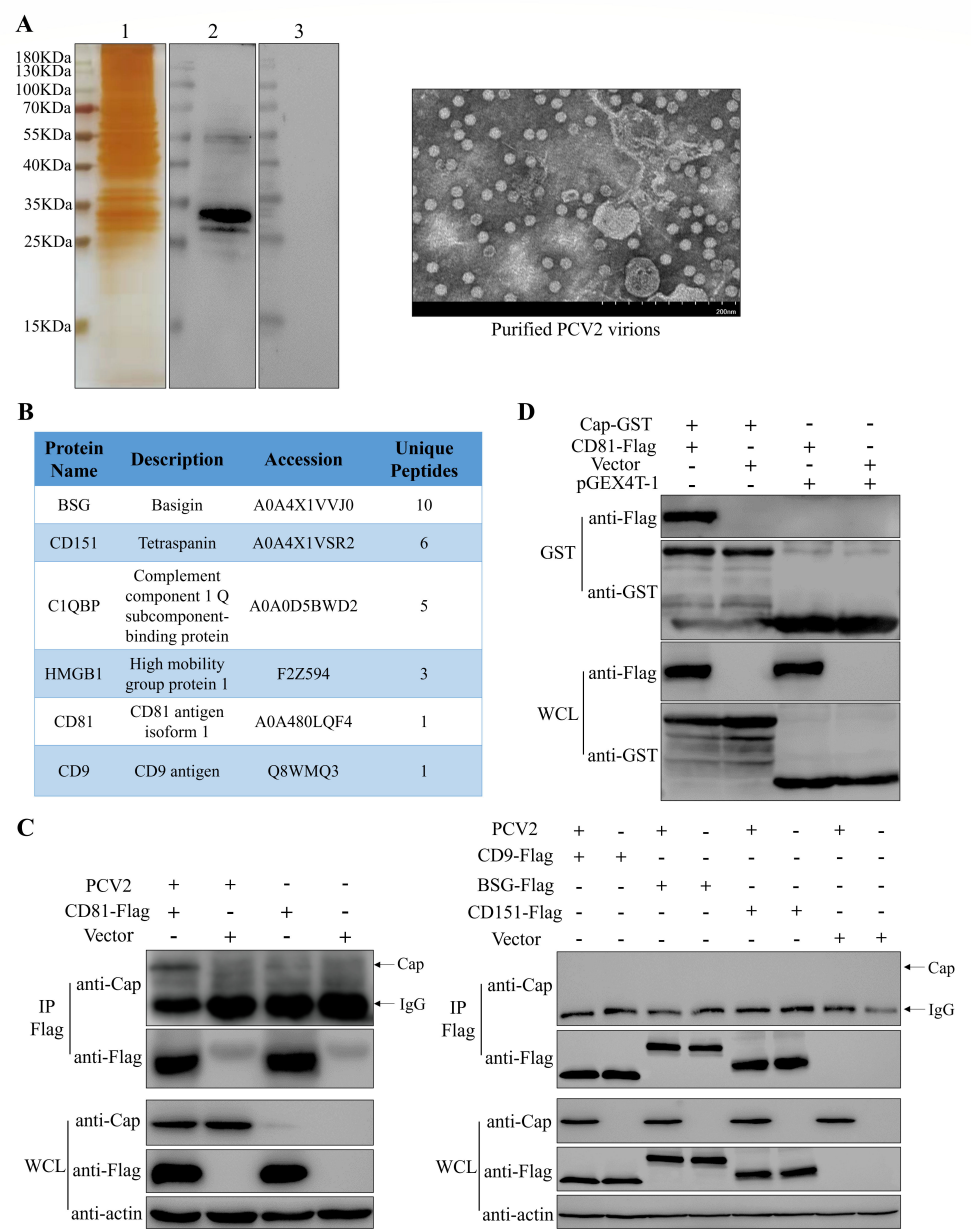
Identification of CD81 as a host protein associated with PCV2 infection via VOPBA. (**A**) Virus overlay protein-binding assay. Lane 1, SDS-PAGE gel silver staining results; lane 2, Western blot results of PCV2 virion-binding protein bands; lane 3, Western blot results of PCV2 virion unbound control group. The purified PCV2 virion was observed with transmission electron microscopy. (**B**) Bioinformatics of some proteins in mass spectrometry analysis results. (**C**) Co-immunoprecipitation identifies the interaction between PCV2 and the candidate proteins in mass spectrometry data. Arrows indicate Cap band and IgG band, respectively. (**D**) GST pull-down experiment identifies the direct interaction between CD81 and recombinant Cap proteins. The vector is the pCAGGS empty control plasmid.

Cap, as the sole structural protein of PCV2, plays a pivotal role during the virus infection. To identify the cellular receptor-related proteins against PCV2 within PK-15 cells, a VOPBA was employed using purified PCV2 virions (Fig. 1A) and anti-Cap monoclonal antibody (mAb) as a probe. As shown in Fig. 1A, several distinct protein bands ranging from 25 to 35 kDa were detected by both silver staining and Western blot with anti-Cap mAb, suggesting that PCV2 were specifically bonded to the 25 ~ 35 kDa host proteins. Mass spectrometry analysis of these specific protein bands revealed a total of 1,225 proteins. Comprehensive information on all proteins is available in the UniProt database. Figure 1B lists some host proteins that may be or have been reported to be associated with PCV2 infection in this mass spectrometry data, such as C1QBP and HMGB1 (32, 33). Based on protein molecular weight, cellular sublocations, and potential biological functions, we selected certain proteins from the mass spectrometry data for protein interaction validation. Interactions between these host proteins and PCV2 virus were detected by co-immunoprecipitation assay (Co-IP), and only CD81 was shown to bind the Cap protein (Fig. 1C). GST pull-down assays with prokaryotically expressed GST-tagged Cap protein (Cap-GST) and cellular overexpressed Flag-tagged CD81 protein (CD81-Flag) demonstrated a consistent result (Fig. 1D).

### CD81 promotes PCV2 replication

**Fig 2:**
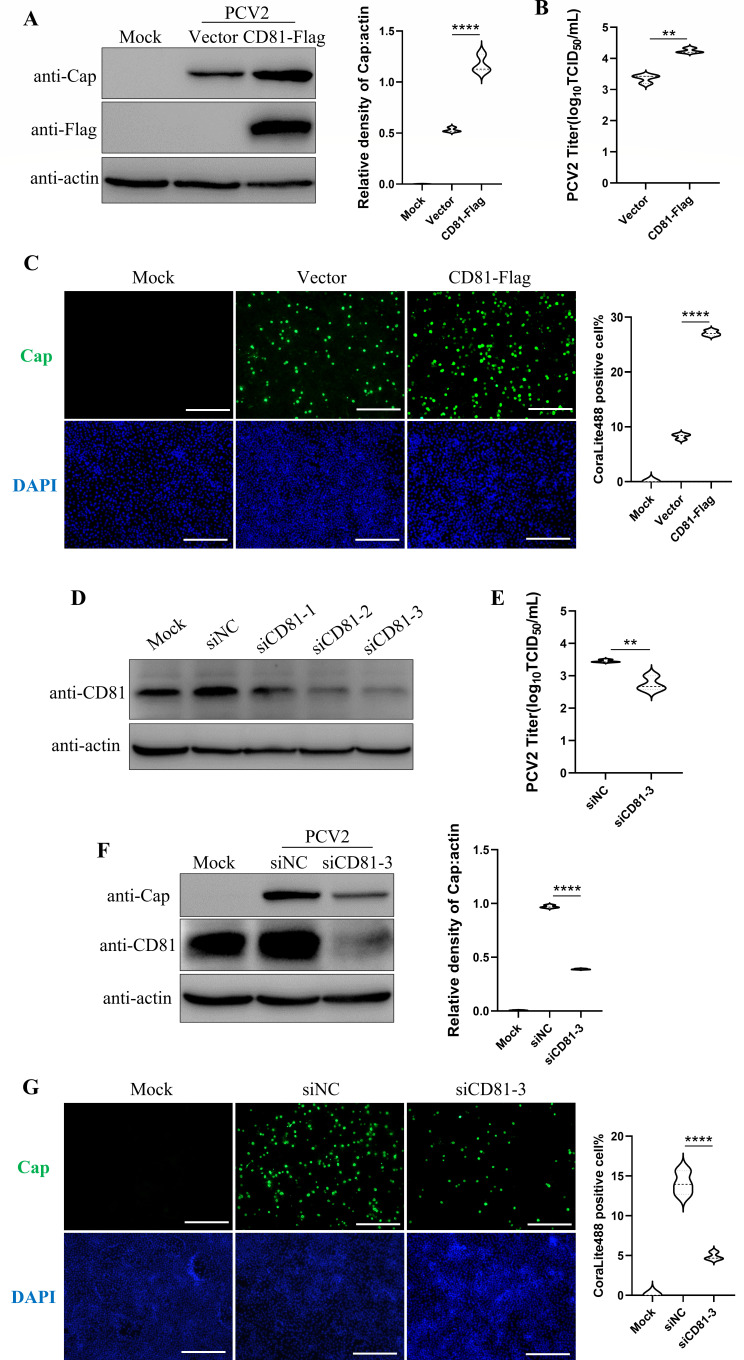
CD81 promotes PCV2 replication. (**A**) pCAGGS-CD81-Flag recombinant plasmids were transfected into PK-15 cells, followed by inoculation with 0.1 MOI PCV2. After 24 h of infection (hpi), Western blot was conducted to detect the expression levels of corresponding proteins. The protein bands were quantified using NIH ImageJ software. (**B**) TCID_50_ assay was performed to determine the influence of overexpressing CD81 on the progeny of PCV2. (**C**) Indirect IFA was conducted to assess the infection status of PCV2 on PK-15 cells after overexpression of CD81. Scale bar, 50 µm. Quantification of CoraLite488 and DAPI (4',6-diamidino-2-phenylindole) fluorescence-positive cells was performed using the ImageJ plugin, respectively. The proportion of PCV2-positive cells relative to the total number of cells within the field of view was statistically analyzed. The vector in A–C is the pCAGGS control plasmid. (**D**) Western blot was conducted to detect the interference effect of CD81 siRNA. siNC (siRNA negative control) is the negative-control interference fragments. (**E–G**) RNA interference. PK-15 cells transfected with CD81 siRNA were infected with 0.1 MOI PCV2 for 36 h. Western blot was conducted to detect the expression levels of PCV2 Cap and endogenous CD81 (**F**). The protein bands were quantified using ImageJ software. TCID_50_ assay was conducted to determine the influence of silencing CD81 gene on the progeny PCV2 (**E**). IFA was performed to evaluate the infection of PCV2 on PK-15 cells after silencing CD81 gene. Also, quantification of CoraLite488 and DAPI fluorescence-positive cells was performed using the ImageJ plugin, respectively. The proportion of PCV2-positive cells was statistically analyzed. (**G**). Scale bar, 50 µm. Data represent the mean ± SD of three independent replicate experiments. *, *P* < 0.05; **, *P* < 0.01; ***, *P* < 0.001.

To explore the effect of CD81 on PCV2 infection, PK-15 cells were transfected with the pCAGGS-CD81-Flag plasmid for 24 h and inoculated with PCV2 (0.1 multiplicity of infection [MOI]) for another 24 h. Figure 2A and B demonstrated that CD81 overexpression promoted PCV2 replication as both viral Cap protein expression and infectious viral particles were significantly up-regulated. Consistently, immunofluorescence assays (IFA) also showed an increased number of PCV2-infected cells upon CD81 overexpression (Fig. 2C). Meanwhile, three siRNAs (Small interfering RNA) targeting CD81 gene were designed and used to knock down CD81 expression in PK-15 cells, and siCD81-3 showed a best knockdown effectivity (Fig. 2D). PK-15 cells were transfected with siCD81-3 and infected with PCV2, and the cells were collected at 36 hpi for Western blot, TCID_50_(50% tissue culture infectious dose), and IFA analysis. As shown in Fig. 2E through G, knockdown of CD81 significantly suppressed PCV2 replication. Collectively, CD81 promoted PCV2 replication in PK-15 cells.

### CD81 is involved in PCV2 internalization into PK-15 cells

**Fig 3:**
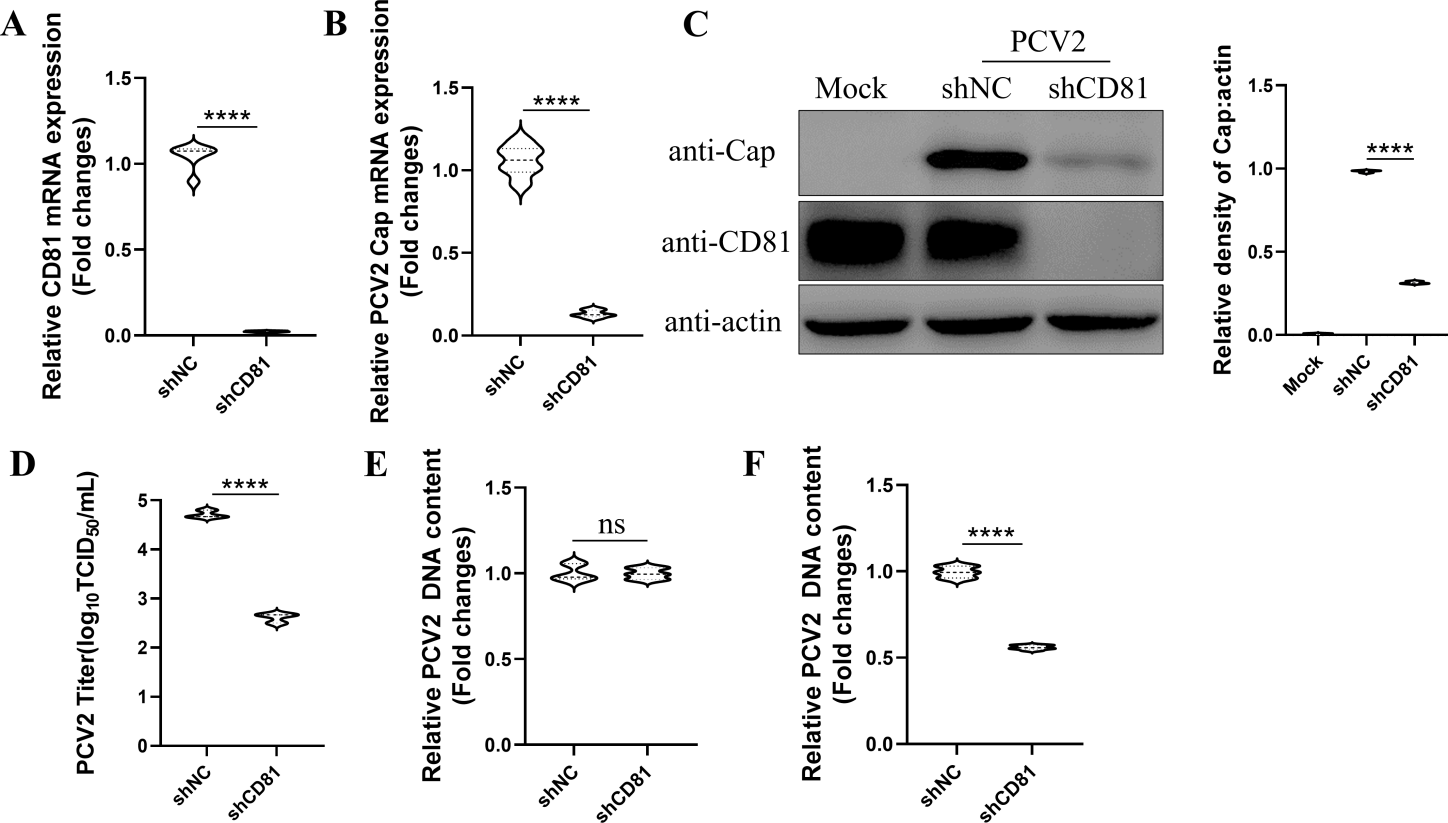
CD81 is involved in PCV2 internalization into PK-15 cells. (**A–C**) CD81 knockdown (shCD81) and negative-control shRNA (shNC) PK-15 cells were infected with 0.1 MOI PCV2. After 36 h, RNA and protein samples were collected. qPCR was performed to detect the expression levels of CD81 mRNA (**A**) and PCV2 Cap mRNA (**B**). Western blot was conducted to detect the expression levels of PCV2 Cap protein and endogenous CD81. The protein bands were quantified using ImageJ software (**C**). TCID_50_ assay was used to determine the viral titer of PCV2 (**D**). (**E**) The virus adsorption assay. CD81 knockdown and shNC PK-15 cells were inoculated with 5 MOI PCV2 and incubated for 1 h at 4°C. DNA samples were extracted, and qPCR was performed to detect the relative content of PCV2, with mtDNA (Mitochondrial DNA) as an internal control. The y-axis represents the fold change in viral DNA content between the experimental and the control groups, calculated using the 2^-ΔΔCT^ formula based on the CT values of the target gene Cap and the reference gene mtDNA (Mitochondrial DNA). (**F**) The virus internalization assay. CD81 knockdown and shNC PK-15 cells were inoculated with 5 MOI PCV2 and incubated for 1 h at 4°C, and then transferred to 37°C for an additional 2 h. DNA samples were extracted, and qPCR was performed to detect the relative content of PCV2, with mtDNA as an internal control. The y-axis represents the fold change in viral DNA content between the experimental and control groups, calculated using the 2^-△△CT^ formula based on the CT values of the target gene Cap and the reference gene mtDNA. Data represent the mean ± SD of three independent replicate experiments. *, *P* < 0.05; **, *P* < 0.01; ***, *P* < 0.001.

As CD81 is located on the cell membrane, it is suspected that CD81 might up-regulate PCV2 infection at the stages of virus attachment or internalization. Thus, CD81 knockdown PK-15 cells were firstly generated using a lentiviral system with shRNA (Short hairpin RNA) specifically targeting CD81 gene (shCD81) (Fig. 3A). Knockdown of CD81 with shCD81 successfully suppressed PCV2 infection as demonstrated by the significantly reduced levels of Cap mRNA, protein expression, and the viral TCID_50_ (Fig. 3B and D). To determine the specific stage at which CD81 regulates PCV2 invasion, the virus attachment and internalization experiments were then conducted. Interestingly, knockdown of CD81 showed no effect on PCV2 attachment onto PK-15 cells but significantly suppressed viral internalization into the cells (Fig. 3E and F).

### The key regions involved in the interaction between CD81 and PCV2 Cap protein

**Fig 4:**
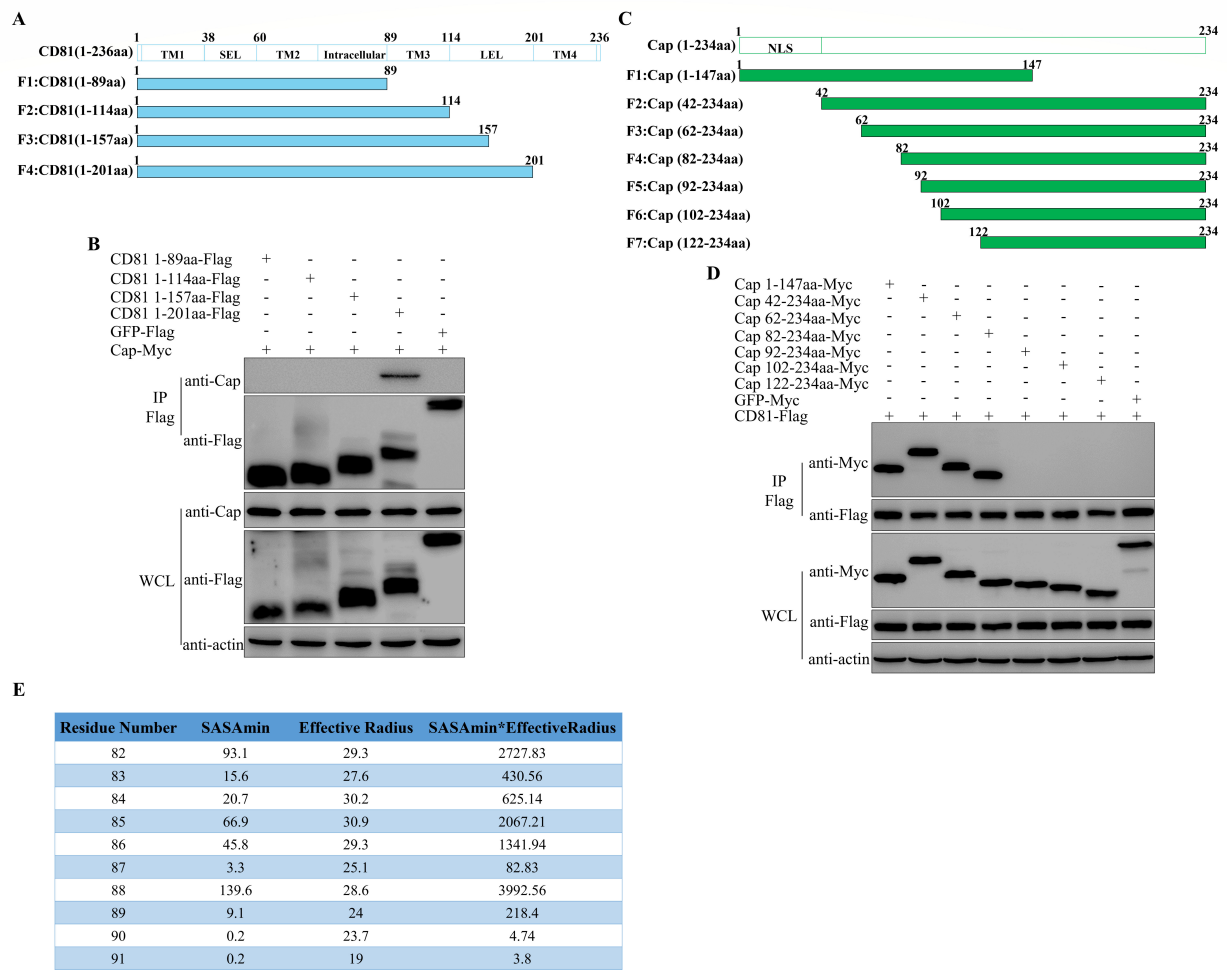
The key regions involved in the interaction between CD81 and PCV2 Cap protein. (**A**) Schematic diagram of CD81 protein truncation construction. (**B**) Co-IP was performed to identify the interaction between PCV2 Cap protein and the various truncated forms of CD81 in human embryonic kidney (HEK)-293T cells. (**C**) Schematic diagram of Cap protein truncation construction. (**D**) Co-IP was performed to identify the interaction between CD81 and the various truncated forms of PCV2 Cap protein in HEK-293T cells. (**E**) SASA and SASAmin * EffectiveRadius value of residues 82–91 in the PCV2 Cap 3D structure 3JCI were evaluated by using the Annotations tool in the Virus Particle Explorer Server.

CD81 consists of four transmembrane domains, one intracellular domain, and two extracellular domains. To elucidate the key regions of CD81 involved in its interaction with PCV2 Cap protein, four truncated forms of CD81 were constructed as illustrated in Fig. 4A. Co-IP revealed that CD81 requires the intact extracellular domain (LEL) to interact with PCV2 Cap protein (Fig. 4B). The functional regions of Cap protein to interact with CD81 was also explored with the Cap truncations. As shown in Fig. 4D, these experiments suggested that aa 82–91 of Cap protein are involved in binding to CD81. Also, several amino acids within residues 82–91 exhibit high solvent-accessible surface area (SASA) and SASAmin * EffectiveRadius values (Fig. 4E). It suggests that these amino acids may be located on the exterior surface of the PCV2 virus particle.

### CD81 LEL is an antiviral target against PCV2 infection

**Fig 5:**
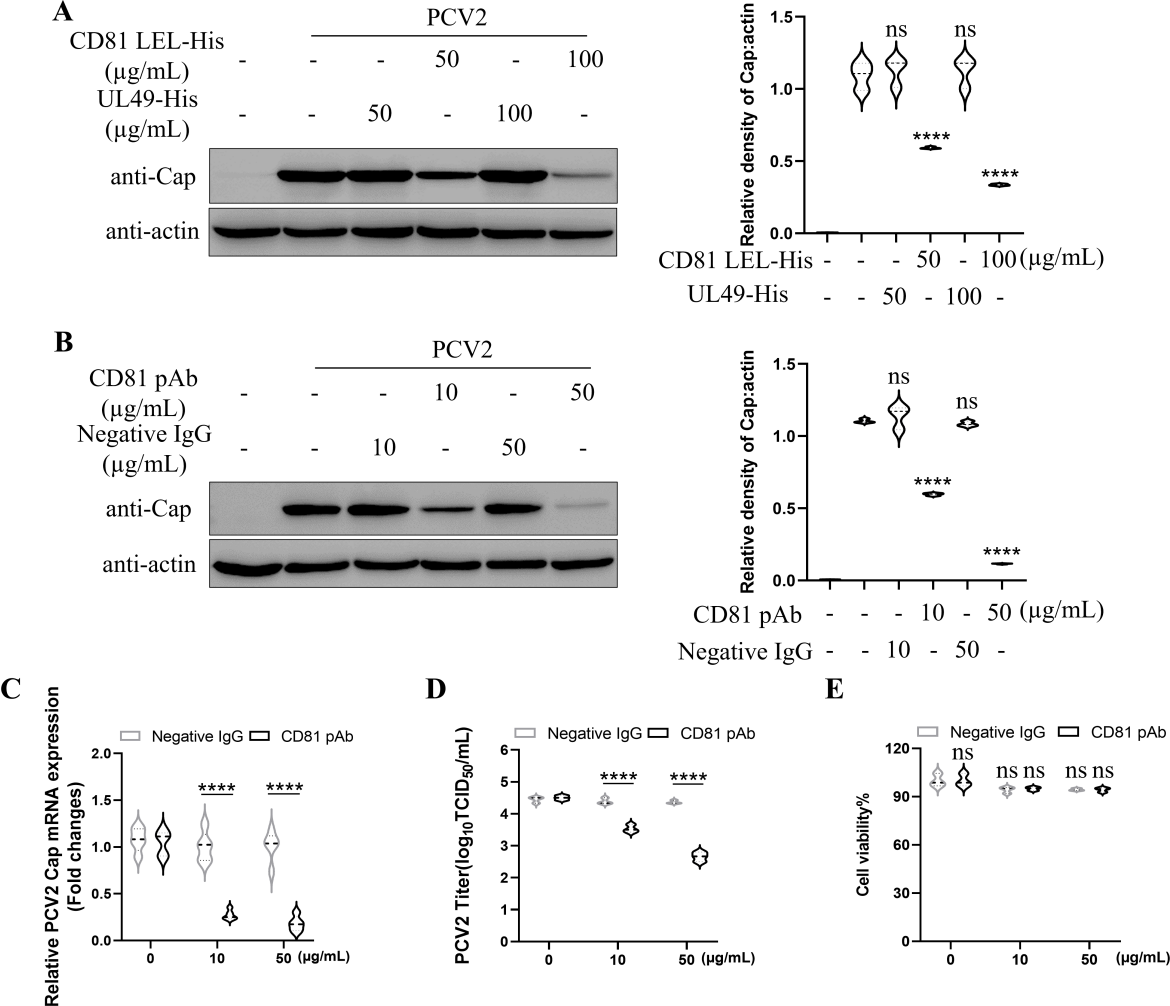
CD81 LEL is an antiviral target against PCV2 infection. (**A**) Inhibition of PCV2 infection by recombinant CD81-LEL protein. PK-15 cells were infected with PCV2 mixed with different concentrations of recombinant CD81-LEL protein. Western blot was performed to detect the expression levels of PCV2 Cap protein. PRV UL49 protein served as an unrelated control group. The protein bands were quantified using ImageJ software. (**B–E**) Anti-CD81 LEL pAb blocking experiments. PK-15 cells pretreated with the pAb against CD81 LEL were infected with PCV2. Western blot and qPCR were performed to detect the levels of PCV2 Cap protein (**B**) and mRNA (**C**). The protein bands were quantified using ImageJ software. TCID_50_ assay was conducted to detect the progeny PCV2 (**D**). Meanwhile, PK-15 cells were treated with anti-CD81 LEL pAb or negative IgG at various concentrations as shown. At 2 days post-treatment, cell viability was analyzed by using CCK-8 assay kit following the manufacturer’s instruction (**E**). Data represent the mean ± SD of three independent experiments. *, *P* < 0.05, **, *P* < 0.01, ***, *P* < 0.001.

As demonstrated above, CD81 LEL region interacted with Cap protein. To investigate whether the CD81 LEL is crucial for PCV2 infection and to identify an antiviral strategy, PCV2 was incubated with different concentrations of prokaryotically expressed recombinant protein CD81 LEL-His at 37°C for 1 h, followed by inoculation in PK-15 cells with this mixture. The cells were collected at 36 h and analyzed by Western blot. As shown in Fig. 5A, incubation with CD81 LEL-His significantly suppressed PCV2 infection in a dose-dependent manner. Moreover, the polyclonal antibodies against CD81 LEL (CD81 LEL-pAb) were prepared and used to treat PK-15 cells before PCV2 infection. Western blot revealed that the blocking of CD81 LEL effectively inhibited PCV2 infection in a dose-dependent manner (Fig. 5B through D). Meanwhile, treatment with CD81 LEL-pAb showed no cytotoxic effect on PK-15 cells (Fig. 5E). These findings collectively indicated that the host protein CD81 could be an antiviral target against PCV2 infection

### Syndecan-1 collaborates with CD81 to facilitate PCV2 infection

**Fig 6:**
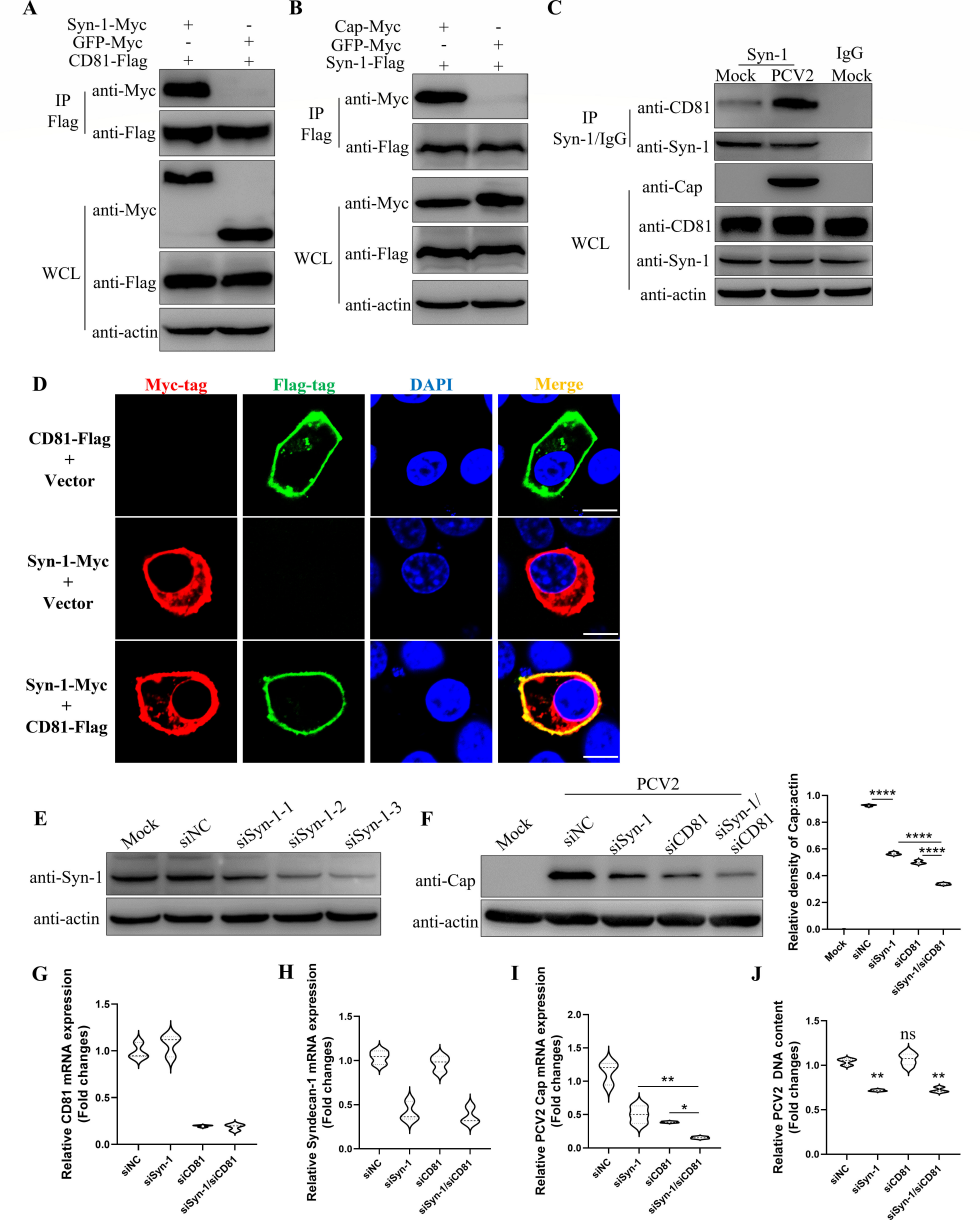
Syndecan-1 collaborates with CD81 to facilitate PCV2 infection. (**A**) The interaction between CD81 and Syndecan-1 was detected with Co-IP. (**B**) The interaction between PCV2 Cap protein and Syndecan-1 was detected with Co-IP. (**C**) The endogenous CD81 and Syndecan-1 were detected in PK-15 cells with Co-IP. PK-15 cells were infected with PCV2 for 36 h, lysed, and immunoprecipitated with anti-Syndecan-1 antibody. The whole-cell lysates and immunoprecipitated products were analyzed by Western blot. (**D**) Immunofluorescence confocal microscopy was used to detect co-localization of CD81 and Syndecan-1 at the cell membrane. Scale bar, 5 µm. (**E**) Western blot was conducted to detect the interference effect of three siRNAs targeting Syndecan-1. (**F–J**) PK-15 cells transfected with the siRNA targeting CD81 and Syndecan-1 were infected with 0.1 MOI PCV2 or 5 MOI PCV2. Western blot was performed to detect the expression levels of PCV2 Cap protein, and the protein bands were quantified using ImageJ software (**F**). qPCR was performed to detect the expression levels of CD81 mRNA (**G**), Syndecan-1 mRNA (**H**), PCV2 Cap mRNA (**I**), and the relative content of adsorbed PCV2, with mtDNA (Mitochondrial DNA) as an internal control (**J**). The y-axis represents the fold change in viral DNA content between the experimental and control groups, calculated using the 2^-ΔΔCT^ formula based on the CT values of the target gene Cap and the reference gene mtDNA (Mitochondrial DNA). Data represent the mean ± SD of three independent experiments. *, *P* < 0.05, **, *P* < 0.01, ***, *P* < 0.001.

Glycosaminoglycans, such as heparan sulfate and chondroitin sulfate, could serve as viral attachment receptors to facilitate virus invasion into host cells (34–38). Syndecan-1 is a cell surface proteoglycan composed of heparan sulfate and chondroitin sulfate, linking cytoskeleton to extracellular matrix (39, 40). To know the roles of Syndecan-1 in PCV2 replication regulated by CD81, the interactions between Syndecan-1 and CD81 in PK-15 cells infected or not infected with PCV2 were examined. As shown in Fig. 6A and B, Co-IP results demonstrated that Syndecan-1 could interact with both CD81 and PCV2 Cap protein. Moreover, PCV2 infection reinforced the interaction between CD81 and Syndecan-1 (Fig. 6C), suggesting that PCV2 may utilize this interaction to promote its own infection. Also, CD81 and Syndecan-1 were co-localized on the cell membrane (Fig. 6D). Furthermore, silencing Syndecan-1 reduces the adsorption of PCV2 and inhibits its infection. And the concurrent silencing of both CD81 and Syndecan-1 exacerbates the inhibition of PCV2 replication (Fig. 6E through J). It indicated that Syndecan-1 collaborated with CD81 to facilitate PCV2 infection.

### CD81 mediates PCV2 internalization by activating RhoA

**Fig 7:**
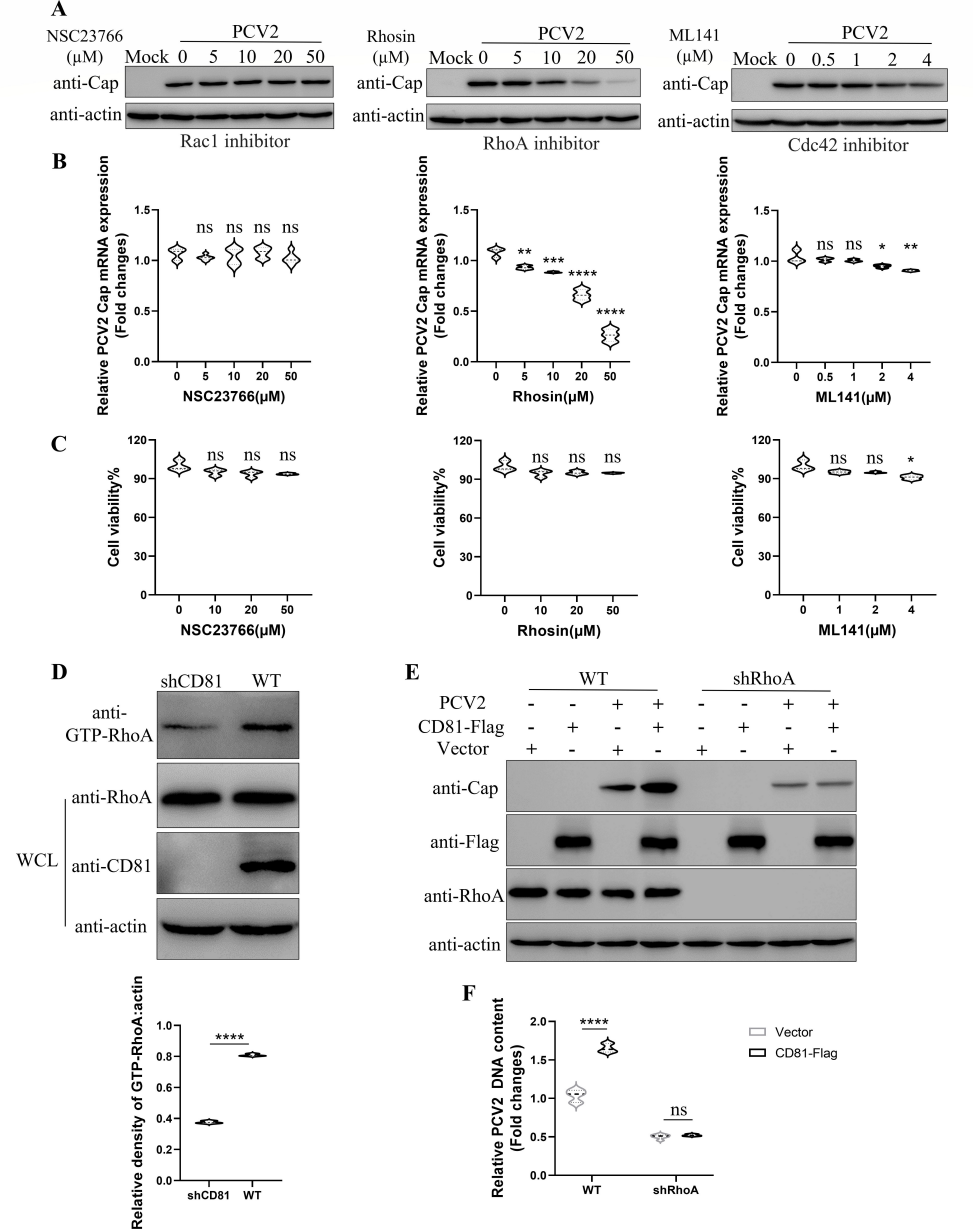
CD81 mediates PCV2 internalization by activating RhoA. (**A and B**) The effects of small GTPase inhibitors on PCV2 infection. PK-15 cells were pretreated with different concentrations of inhibitors at 37°C for 1 h, followed by infection with 0.1 MOI PCV2. After 24 h, the cell protein and RNA samples were collected. Western blot was performed to detect the expression of PCV2 Cap protein (**A**) and qPCR was conducted to determine the expression of PCV2 Cap mRNA (**B**). (**C**) Cell viability. PK-15 cells were treated with different small GTPase inhibitors at various concentrations as shown. At 2 days post-treatment, cell viability was analyzed by using CCK-8 assay kit following the manufacturer’s instruction. Data represent the mean ± SD of three independent experiments. (**D**) GST pull-down assay was utilized to compare the differences in the levels of active RhoA between shCD81 and wild-type (WT) PK-15 cells. The protein bands were quantified using ImageJ software. (**E and F**) Overexpression experiment to identify the role of RhoA in the regulation of PCV2 infection by CD81. WT PK-15 cells and RhoA knockdown (shRhoA) PK-15 cells were transfected with CD81-Flag plasmid, followed by infection with 0.1 MOI or 5 MOI PCV2.Western blot was performed to detect the expression levels of PCV2 Cap protein (**E**). qPCR was performed to detect the relative content of internalized PCV2, with mtDNA (Mitochondrial DNA) as an internal control (**F**). The y-axis represents the fold change in viral DNA content between the experimental and control groups, calculated using the 2^-ΔΔCT^ formula based on the CT values of the target gene Cap and the reference gene mtDNA (Mitochondrial DNA). Data represent the mean ± SD of three independent replicate experiments. *, *P* < 0.05; **, *P* < 0.01; ***, *P* < 0.001.

Small guanosine triphosphatases, also known as small GTPases, have been reported to be associated with PCV2 infection (20). Meanwhile, CD81 as a central regulator in the infection of human hepatocytes by HCV, has been shown to augment the levels of activated Rho GTPases during the viral infection process, thereby facilitating HCV infection (41). To investigate whether the small GTPases are involved in the CD81-mediated PCV2 internalization, PK-15 cells were pretreated with the inhibitors against Rac1, RhoA, and Cdc42, followed by infection with PCV2. As shown in Fig. 7A and B, RhoA inhibitor (Rhosin) suppressed PCV2 replication in a dose-dependent manner, while Rac1 inhibitor (NSC23766) and Cdc42 inhibitor (ML141) showed no or slight effect on PCV replication, indicating that RhoA was associated with PCV2 replication.

The RhoA can cycle between an inactive and active GTP-bound state within cells, thereby regulating various physiological processes (42). Rhotekin, as an effector protein of the small GTPase family, can specifically bind to the activated form of RhoA (GTP-bound state) (43). The effect of CD81 on RhoA activation was detected by analysis of the expression of RhoA and GTP-RhoA in wild-type (WT) and shCD81 PK-15 cells. The results showed no difference of RhoA expression levels in WT and shCD81 PK-15 cells. However, the GTP-RhoA pulled down by Rhotekin in shCD81 PK-15 cells was significantly lower in WT cells (Fig. 7D), indicating that CD81 facilitated RhoA activation.

To verify further whether CD81 promoted PCV2 internalization and replication through activating RhoA, RhoA knockdown (shRhoA) PK-15 cells were generated and used to detect the effect of CD81 on PCV2 infection. As shown in Fig. 7E and F, overexpression of CD81 promoted PCV2 internalization and replication in WT PK-15 cells, but not in shRhoA PK-15 cells. These results suggested that CD81 promoted PCV2 internalization and replication through activating RhoA.

**Fig 8:**
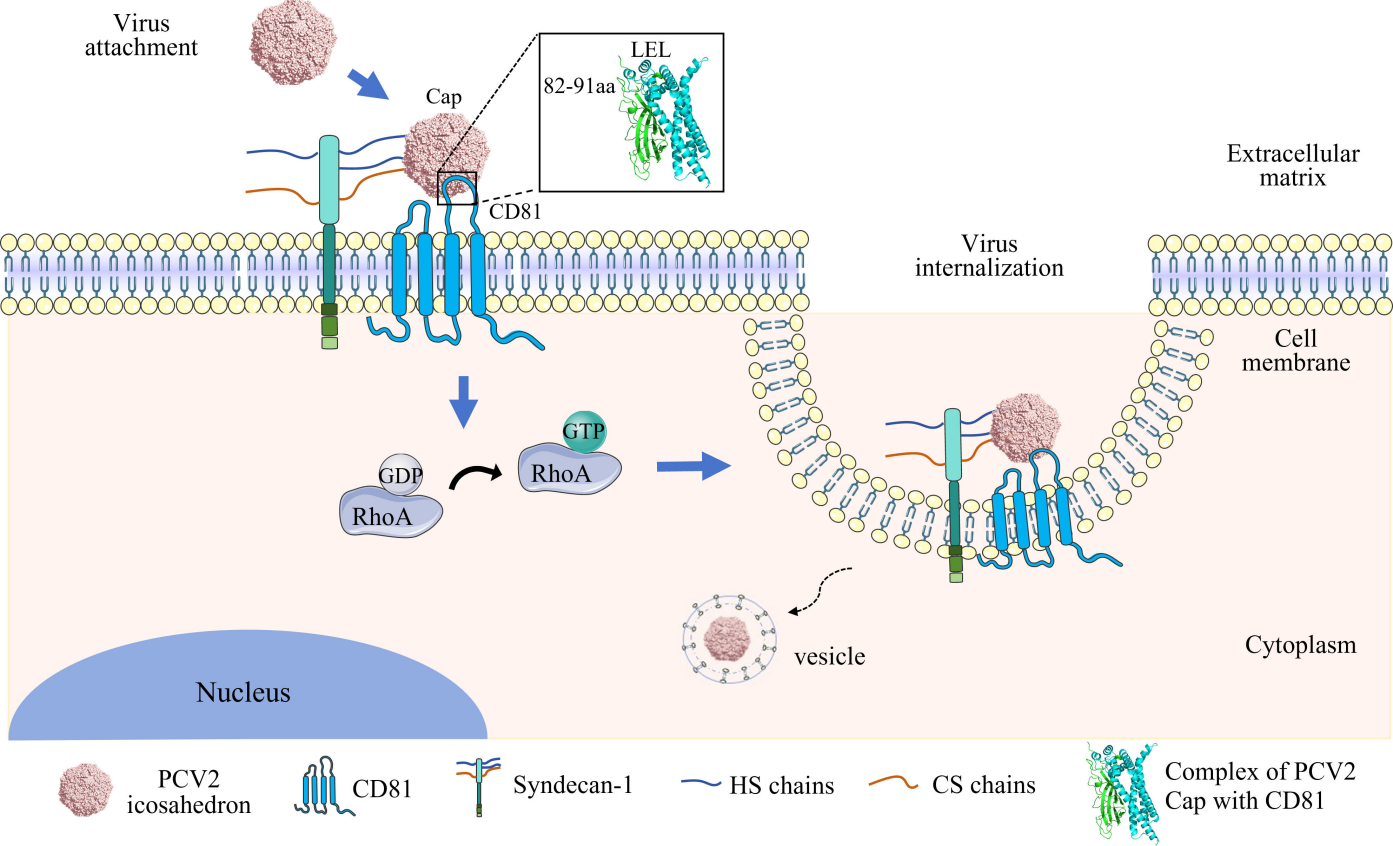
Model of CD81-mediated PCV2 invasion in PK-15 cells. During PCV2 attachment to PK-15 cells, the CD loop region (82–91aa) of the virus Cap protein interacts with the LEL domain of CD81 and Syndecan-1. The picture of docking of PCV2 Cap with CD81 was prepared with the SWISS-MODEL website and GRAMM Docking Web. CD81 also enhances the active level of intracellular RhoA and facilitates PCV2 internalization, resulting in enhancing PCV2 infection in PK-15 cells. This model was created by the author for the first time. Parts of the figure were drawn by using pictures from Servier Medical Art (https://smart.servier.com). Servier Medical Art is licensed under CC BY 4.0 (https://creativecommons.org/licenses/by/4.0/).

The model of CD81-mediated PCV2 invasion in PK-15 cells is shown in Fig. 8.

## DISCUSSION

As a transmembrane protein predominantly expressed on the cell membrane, CD81 is not only indispensable for cellular biology but also plays pivotal roles in the life cycles of various pathogens (23, 29, 44–46). As an organizer of plasma membrane structures, it forms ‘Tetraspanin-enriched microdomains” (TEMs) with other tetraspanins, which serve as regions for the attachment of numerous signaling molecules or receptors. CD81 contains two extracellular loop domains: a LEL and a SEL, with the LEL being crucial for the recognition of most ligands, antibodies, and pathogens, thus essential for the corresponding biological functions of CD81 (47). For instance, during HCV infection, the virus initially utilizes its E2 glycoprotein to interact with the LEL domain of CD81, allowing CD81 to act as a viral receptor and mediate its entry into hepatocytes (27). Antibodies against CD81 or recombinant CD81-LEL proteins can inhibit HCV pseudoparticles and HCV cell culture-derived virus infections (48–50). Additionally, some studies have shown that during influenza virus infection, CD81 is specifically recruited to the sites of influenza virus assembly and budding, aiding in viral egress and promoting virus proliferation (29). These studies indicate that the tetraspanin CD81 plays corresponding roles at different stages of viral life cycles. In this study, we found and identified CD81 as a host protein associated with PCV2 infection using VOPBA and mass spectrometry analysis. Subsequent protein immunoprecipitation and GST pull-down assays confirmed the interaction between CD81 and PCV2 Cap protein, with the interaction region located in the LEL domain of the CD81 molecule. Silencing CD81 expression significantly impairs PCV2 internalization and replication in PK-15 cells, indicating that CD81 plays a critical role in the process of PCV2 invasion into the cells.

PCV2 infection in epithelial cells is independent of clathrin-, caveolin-, and dynamin-mediated endocytic pathways but is associated with pathways mediated by Rho GTPase proteins (20). Here, our results showed that treatment with the inhibitor targeting the RhoA protein significantly inhibited PCV2 infection in PK-15 cells. Moreover, CD81, in this context, facilitates the internalization and replication of PCV2 by enhancing the activation level of RhoA protein. This may be attributed to the rearrangement of cytoskeletal proteins regulated by RhoA protein (51, 52). However, the precise mechanisms underlying this process require further investigation.

Heparan sulfate proteoglycans can serve as attachment receptors, mediating the binding of various viruses to the surface of target cells (34–38). PCV2 also can utilize heparan sulfate and chondroitin sulfate B as its attachment receptors to adhere to host cell surfaces (15). Syndecan-1, a transmembrane proteoglycan, primarily comprises heparan sulfate and chondroitin sulfate (39, 40, 53). It has been reported that Syndecan-1 can act as an attachment receptor for viruses such as severe acute respiratory syndrome coronavirus 2 and HCV (54, 55), facilitating viral invasion, while also regulating the egress of HSV-1 (Herpes Simplex Virus Type 1)‌‌ from infected cells (56, 57). In this study, Co-IP experiments results demonstrated the interaction between Syndecan-1, CD81, and PCV2 Cap protein. Additionally, RNA interference experiments revealed that silencing Syndecan-1 could affect PCV2 attachment in PK-15 cells. Concomitant knockdown of CD81 and Syndecan-1 genes inhibited PCV2 infection more effectively than knockdown of either protein alone, indicating that CD81 and Syndecan-1 co-operate to promote PCV2 infection in PK-15 cells. In fact, a significant functional aspect of multiligand proteoglycans is their ability to bind to other types of receptors, thereby mediating signal transduction through these receptors (40). Consequently, we hypothesize that CD81 and its associated TEMs likely provide a platform for the recruitment of Syndecan-1. When present within TEMs, Syndecan-1 may more effectively recognize pathogens and transduce signals through CD81 or other proteins. Simultaneously, given that Syndecan-1 may not be entirely localized within TEMs, knockdown of the CD81 gene is unlikely to exert a significant impact on the adsorption of PCV2.

Tetraspanin CD81 can be used as the receptor of HCV and mediate the viral entry into hepatocytes (27). In order to investigate whether the CD81 LEL region is crucial for PCV2 infection and to identify an antiviral strategy, we examined the inhibition efficacy of the purified recombinant protein CD81 LEL (aa 114–201) and anti-CD81-LEL antibody. The results showed that incubation with the CD81 LEL-His or polyclonal antibodies against CD81 LEL effectively suppressed PCV2 infection in a dose-dependent manner. But the dose of CD81 LEL-His for partial inhibition of PCV2 infection was significantly higher than that reported in HCV (50). The possible reasons might be the auxiliary receptor rules of CD81 in PCV2 infection in PK-15 cells or the biological activity of recombinant protein CD81 LEL-His. Hsu et al. used a soluble human CD81 and found it could inhibit 50% of HCV infections at about 1 µg/mL, and >90% of infections at 5 µg/mL. Here, the pig CD81 LEL-His was expressed in *Escherichia coli* in the form of inclusion bodies and purified by affinity chromatography in our lab (data not shown here). Next, the pig CD81 LEL should be expressed in a soluble form by using other expression systems and used to examine the inhibiting efficacy of PCV2 infection in the future.

In conclusion, CD81 regulates PCV2 internalization and replication in PK-15 cells by interacting with PCV2 Cap protein and Syndecan-1, and activation of RhoA. A polyclonal antibody against CD81 LEL domain significantly blocks the virus infection in the cells. This not only enriches and refines the understanding of the infection mechanism of PCV2 but also provides a novel host target for the development of anti-circovirus therapeutics.

## MATERIALS AND METHODS

### Cells, viruses, and plasmids

Human embryonic kidney (HEK)-293T and porcine kidney cell 15 were maintained in Dulbecco’s modified Eagle’s medium (DMEM, Gibco) supplemented with 10% fetal bovine serum, 100 U/mL penicillin, and 100  µg/mL streptomycin. All cells were kept in a humidified 5% CO_2_ incubator at 37°C.

The PCV2 SH strain (GenBank: AY686763.1) was isolated and preserved by our laboratory from PCV2-positive samples.

The CD81 gene (GenBank: JQ410171.1), CD9 gene (GenBank: NM_214006.1), BSG gene (GenBank: NM_001123086.1), CD151 gene (GenBank: NM_001243865.1), and Syndecan-1 gene (GenBank: NM_001243190.1) were amplified from PK-15 cells by RT-PCR and then cloned into the pCAGGS vector fused with Flag or Myc tag. The Cap gene was amplified from the laboratory-stored pVAX-SynCap plasmid and cloned into the pCAGGS vector after fusion with Myc tag. These recombinant plasmids were designated as CD81-Flag, CD9-Flag, BSG-Flag, CD151-Flag, Syn-1-Flag, Syn-1-Myc, and Cap-Myc, respectively. The truncated plasmids of Cap and CD81 genes were respectively amplified using Cap-Myc or CD81-Flag as templates.(Table 1)

**TABLE 1:** Primer sequences for amplification and construction of the candidate protein genes and their truncated plasmids

Primer	Sequence (5′→3′)
CD81-Flag-F:	CTGAATTCATGGGGGTAGAGGGCTGCAC
CD81-Flag-R:	CCCTCGAGTCACTTATCGTCGTCATCCTTGTAATCGTACACGGAGCTGTTCC
CD9-Flag-F:	CGGAATTCATGCCGGTCAAAGGAGGCACTAAG
CD9-Flag-R:	GGCTCGAGCTACTTATCGTCGTCATCCTTGTAATCGACCATCTCTCGGCTCCGAC
BSG-Flag-F:	CGGAATTCATGGCGGACGTGAGACTCG
BSG-Flag-R:	CCCTCGAGTCACTTATCGTCGTCATCCTTGTAATCGCTGGCATTTCTCTGGCGAACGT
CD151-Flag-F:	TAGGTACCATGGGCGAATTCGGCGAGAAGG
CD151-Flag-R:	CCCTCGAGTCACTTATCGTCGTCATCCTTGTAATCGTAGTGCTCCAGCTTCAGGCTCT
Syn-1-Flag-F:	ATGGTACCATGAGGCGCGCGGCGTTTTG
Syn-1-Flag-R:	CCCTCGAGTCACTTATCGTCGTCATCCTTGTAATCGGCGTAGAACTCCTCCTGCCTGC
Syn-1-Myc-F:	ATGGTACCATGAGGCGCGCGGCGTTTTG
Syn-1-Myc-R:	CCCTCGAGTCACAGATCCTCTTCAGAGATGAGTTTCTGCTCGGCGTAGAACTCCTCC
Cap(1–147aa)-Myc-F:	TCGGTACCGCCACCATGACCTACCCACGCCGGC
Cap(1–147aa)-Myc-R:	ACCTCGAGTCACAGATCCTCTTCAGAGATGAGTTTCTGCTCCCGGCTGGAGTAATTGAC
Cap-Myc-F:	CGGGATCCGCCACCATGACCTACCCACGCCGGC
Cap(42–234aa)-Myc-F:	TCGGTACCGCCACCATGAACGGCATCTTCAATACC
Cap(62–234aa)-Myc-F:	ATGGTACCGCCACCATGCGGACACCAAGCTGGAATG
Cap(82–234aa)-Myc-F:	AAGGTACCGCCACCATGGGAGGCGGGAGCAACCCCCTG
Cap(92–234aa)-Myc-F:	TCGGTACCGCCACCATGTTCGAGTACTATCGCATC
Cap(102–234aa)-Myc-F:	TCGGTACCGCCACCATGGTGGAGTTCTGGCCATGC
Cap(122–234aa)-Myc-F:	TCGGTACCGCCACCATGATCCTGGACGACAACTT
Cap-Myc-R:	ACCTCGAGTCACAGATCCTCTTCAGAGATGAGTTTCTGCTCCTTGGGATTGAGAGGGGG
CD81 (1–89aa)-Flag-R:	ATCTCGAGTCACTTATCGTCGTCATCCTTGTAATCGCACTGTGACTCCTGGATG
CD81 (1–114aa)-Flag-R:	ATCTCGAGTCACTTATCGTCGTCATCCTTGTAATCGACGAAACCCCAGATGCCGG
CD81 (1–157aa)-Flag-R:	ATCTCGAGTCACTTATCGTCGTCATCCTTGTAATCGCAGCAGTTGAGCGTCTCGT
CD81 (1–201aa)-Flag-R:	ATCTCGAGTCACTTATCGTCGTCATCCTTGTAATCCAGCTTCCCCGAGAAGAGCTC

### Virus proliferation, purification and observation with electron microscopy

PCV2 was inoculated in PK-15 cells at an MOI of 1, and incubated at 37°C for 72 h. Then the cells were subjected to three freeze-thaw cycles, followed by centrifugation at 14,000 × *g* for 30 min at 4°C to collect the supernatant, which was then filtered through a 0.22 µm filter. The filtered liquid was centrifuged at 28,700 × *g* for 4 h at 4°C, and the resulting pellet was suspended in 10 mL of DMEM. Also, the purified PCV2 virion was observed with transmission electron microscopy (CM100, Philips Electron Optics, Zurich, Switzerland). The concentration of viral protein in the suspended solution was determined using the BCA method.

### VOPBA and mass spectrometry analysis

The membrane proteins of PK-15 cells were extracted by using Cell Membrane and Cytosol Protein Extraction Kit (Beyotime, P0033) according to the manufacturer’s instruction. Subsequently, the extracted membrane proteins were subjected to electrophoretic separation using two identical 12% SDS-PAGE gels. Upon completion of electrophoresis, one gel was processed using a Fast Silver Stain Kit (Beyotime, P0017S) according to the manufacturer’s instructions for silver staining, while the other gel was used for protein transfer. The proteins were transferred onto a nitrocellulose (NC) membrane. The NC membrane was then blocked in TBST (Tris-Buffered Saline with Tween-20) buffer (containing 5% skim milk) at 4°C overnight. Next, the NC membrane was transferred to protein-binding buffer (50 mM Tris-HCl, 5 mM CaCl2, 10 mM MgCl2, pH 6.5) and incubated with 10 µg PCV2 particles at 4°C overnight. After washing the NC membrane three times with TBST, it was incubated with specific monoclonal antibody against PCV2 Cap protein (3E5, made in our laboratory) at 4°C overnight. Following binding of PCV2 antibody, the membrane was further incubated with HRP (Horseradish Peroxidase)-conjugated goat anti-mouse IgG at room temperature for 1 h. Finally, the NC membrane was subjected to chemiluminescent imaging analysis.

Based on the imaging results, the protein bands on the NC membrane represent potential host proteins interacting with the PCV2 Cap protein. Corresponding regions on the silver-stained gel were excised, and the excised protein gel strips were placed in double-distilled water. These samples were then sent to Shanghai Applied Protein Technology Co., Ltd. for mass spectrometry analysis (LC-MS/MS, liquid chromatography tandem mass spectrometry).

### Indirect IFA

Using a 96-well cell culture plate as an example, PK-15 cells were seeded on a 96 well plate and then infected with PCV2 for 36 hours, after that the culture medium in each well was discarded. The cells were then washed three times with sterile PBS (Phosphate Buffered Saline). Subsequently, 100 µL of precooled anhydrous ethanol was added to fix the cells at 4°C for 30 min. Following fixation, the cells were washed three times with sterile PBS, and then incubated with 100 µL of primary antibody at 4°C overnight. After washing the cells three times with PBST (Phosphate Buffered Saline with Tween-20‌), 100 µL of CoraLite488-conjugated Goat Anti-Mouse IgG (Proteintech, SA00013-1) was added, and the cells were incubated at 37°C for 1 h. The cell nuclei were stained with DAPI staining solution (Biosharp, BL105A) at room temperature in the dark for 5 min. After three washes with sterile PBS, the cell plate was examined under a fluorescence microscope. Quantification of CoraLite488 and DAPI fluorescence-positive cells under different conditions was performed using the ImageJ plugin, respectively. The proportion of the positive cells relative to the total number of cells within the field of view was statistically analyzed.

### Western blot

To prepare the protein samples for Western blot, the cells were lysed by adding 100 µL of cell lysis buffer (Beyotime, P0013) to each well of 12-well plate and placed on ice for 30 min. Subsequently, the lysates were centrifuged at 14,000 × *g* for 5 min at 4°C, and the supernatants were mixed with 5× loading buffer. After boiling, the proteins were then separated by SDS-PAGE and transferred onto nitrocellulose membranes. Subsequently, the membranes were blocked with PBST containing 5% skim milk at room temperature for 2 h. The blocked membranes were washed three times with PBST for 10 min each, followed by the addition of corresponding primary antibodies (Abmart, M20008, M20002; Proteintech, 66009-1-Ig, 10593-1-AP; CST, 2117T; ABclonal, A5270) and overnight incubation at 4°C. Afterward, the membranes were washed three times with PBST for 10 min each, followed by the addition of HRP-conjugated goat anti-mouse IgG or goat anti-rabbit IgG and incubated at room temperature for 1 h. The membranes were then washed three times with PBST for 10 min each, and ECL substrate was evenly applied onto the membranes. Imaging was performed using a chemiluminescence imaging system.

### Co-IP

To identify the interaction between PCV2 Cap and the candidate proteins in mass spectrometry data (CD81, CD9, BSG, and CD151), co-immunoprecipitation assays were conducted as follows: HEK-293T cells were transfected with CD81-Flag, CD9-Flag, BSG-Flag, CD151-Flag, or pCAGGS empty control vector. After 24 h of transfection, cells were lysed and proteins were extracted. After centrifugation at 4°C, the supernatant was mixed with PCV2 virus, and 80 µL of mixtures were served as the whole-cell lysate (WCL). The remaining mixture was incubated overnight at 4°C with 50 µL Flag magnetic beads (Sigma-Aldrich, M8823). The magnetic beads were then separated from the supernatant using a magnetic stand, washed five times with pre-chilled sterile PBS. Also, the magnetic beads were resuspended in 60 µL of 2× loading buffer and boiled for Western blot analysis. To identify the interaction between PCV2 Cap and CD81 protein, HEK-293T cells were co-transfected with CD81-Flag and Cap-Myc or GFP-Myc plasmids. After 24 h of transfection, the cells were lysed and proteins were extracted. The protein samples were prepared as the above methods and subjected to Western blot analysis.

To identify the interaction between Syndecan-1 and CD81 or PCV2 Cap protein, co-immunoprecipitation assays were conducted by using HEK-293T cells. The cells were co-transfected with CD81-Flag and Syndecan-1-Myc plasmids or Syndecan-1-Flag and Cap-Myc plasmids. After 24 h of transfection, the cells were lysed and proteins were extracted. The protein samples were prepared as the above methods and subjected to Western blot analysis.

### GST pull-down assay

The PCV2 Cap gene was cloned into the pGEX-4T-1 vector and transformed into *Escherichia coli* for prokaryotic expression induced by IPTG (Isopropyl β-D-1-thiogalactopyranoside). Ten milliliters of induced bacterial culture was collected by centrifugation at 14,000 × *g* for 1 min at 4°C. The bacterial pellets were resuspended in 1 mL of sterile PBS and lysed using an ultrasonic cell disruptor. After centrifugation at 14,000 × *g* for 10 min at 4°C, 80 µL of the supernatant was aspirated and mixed with 20 µL of 5× loading buffer, then the mixture was boiled to serve as WCL. Then, the remaining supernatant was collected and added with 40 µL Glutathione MagBeads (Genscript, L00895). After incubation overnight at 4°C with rotation, the magnetic beads were separated from the supernatant using a magnetic stand, washed five times with pre-chilled sterile PBS, and kept at 4°C for further use.

HEK-293T cells were transfected with CD81-Flag plasmid for 24 h, followed by cell lysis and protein extraction. The CD81 protein was mixed with the above prepared Glutathione MagBeads and incubated at 4°C for 3 h with rotation. After another round of magnetic separation and five washes with pre-chilled sterile PBS, the magnetic beads were eluted with 100 µL elution buffer (10 mM GSH, 0.05 M Tris-HCl, pH 8.0), supplemented with 25 µL of 5× loading buffer, boiled, and subjected to Western blot analysis.

### qPCR

According to the gene sequences of PCV2 SH strain (GenBank: AY686763.1) published by NCBI, as well as the gene sequences of porcine CD81 (GenBank: JQ410171.1), porcine Syndecan-1 (GenBank: NM_001243190.1), and porcine actin (GenBank: EU655628.1), corresponding qPCR primers were designed. The primer sequences are listed in Table 2. Following the instructions of AceQ qPCR SYBR Green Master Mix (Vazyme, Q111), the reaction system was prepared in PCR tubes as follows: Primer F (10 µM), 0.4 µL; Primer R (10 µM), 0.4 µL; 2× AceQ qPCR SYBR Green Master Mix, 10 µL; 50× ROX Reference Dye 2, 0.4 µL; template cDNA, 1 µL; ddH_2_O to a final volume of 20 µL. After thorough mixing, PCR amplification was performed with the following program: step 1, pre-denaturation, 95°C, 5 min; step 2, 95°C, 10 s, 60°C, 30 s, repeated for 40 cycles; step 3, melting curve analysis, 95°C, 15 s, 60°C, 1 min, 95°C, 15 s.

**TABLE 2:** qPCR primer sequences

Primer	Sequence (5′→3′)
qCD81-F:	TGATGTTTGTGGGGTTCCTG
qCD81-R:	TGGTCCTTGTTGACGAAACC
qCap-F:	CGCTGGAGAAGGAAAAATGGC
qCap-R:	TTCTGACTGTGGTAGCCTTGAC
qSyndecan-1F:	ACTTCACCTTTGACGTGTCC
qSyndecan-1-R:	TCCCAGCACTTCCTTCCTATC
β-Actin-F:	GACCTGACCGACTACCTCATG
β-Actin-R:	TCTCCTTGATGTCCCGCAC
qmtDNA-F:	AAGACATCGGCACCCTGTAC
qmtDNA-R:	CTGACCTAGTTCAGCGCGAA

### TCID_50_ assay

PK-15 cells were seeded into 96-well plates. When the cell confluence reached 70%–80% monolayer, 100 µL of virus solution, serially diluted by 10-fold, was added to each well with eight replicates for each dilution. After 1.5 h of incubation at 37°C in a cell culture incubator, the virus solution was discarded, and maintenance medium was added for further incubation for 72 h. After 72 h of viral infection, an indirect immunofluorescence assay was performed. The number of wells with and without fluorescence was counted at different virus dilutions, and the TCID_50_ of the virus was calculated according to the Reed-Muench method.

### Evaluation of SASA and SASAmin * EffectiveRadius value of PCV2 Cap residues

By using the PCV2 Cap 3D structure 3JCI (PDB ID) as a template, the SASA and SASAmin * EffectiveRadius value of residues 82–91 in the PCV2 Cap 3D structure were evaluated by using the Annotations tool in the Virus Particle Explorer server.

### Cell viability assay

PK-15 cells were seeded in a 24-well plate. When the cell confluence reached 70%–80% monolayer, different concentrations of ML141 (TargetMol, T2463), Rhosin (MCE, HY-12646), NSC23766 (MCE, HY-15723A) inhibitors, or CD81-LEL pAb were added to each well. Negative control and blank control groups were set up simultaneously. The cells were further incubated for 48 h. Then, 50 µL CCK-8 solution (Beyotime, C0041) was added to each well, and the cell plate was placed in a 37°C incubator, avoiding light, for 2 h. Subsequently, the absorbance of each well was measured at 450 nm using a microplate reader to calculate cell viability.

### RNA interference

According to the gene sequences of porcine CD81 and Syndecan-1, the Genepharma Company designed and synthesized corresponding siRNA fragments and negative-control interference fragments. The siRNA primer sequences are listed in Table 3. PK-15 cells were seeded into 12-well plates. When the cell confluence reached 40%–50% monolayer, the cells were transfected with 50 nM siRNAs per well using the Lipofectamine RNAiMAX (Thermo Fisher Scientific, 13778100) reagent according to the manufacturer’s instructions. After 24 h of transfection, cells were infected with PCV2 at a MOI of 0.1 and incubated at 37°C for 1.5 h, followed by maintenance medium replacement and further incubation for 36 h. After 36 h of viral infection, RNA and protein samples were extracted from the cells for qPCR and Western blot analysis to analyze changes in the mRNA and protein levels of the target genes.

**TABLE 3:** siRNA sequences

Primer	Sequence (5′→3′)
CD81-siRNA-1	Sense: GGAUGAUGUAGAUCAUAAATT
	Antisense: UUUAUGAUCUACAUCAUCCTT
CD81-siRNA-2	Sense: GUCACCAACACCACAAUGATT
	Antisense: UCAUUGUGGUGUUGGUGACTT
CD81-siRNA-3	Sense: GAGGUGCAAUAUUACAACATT
	Antisense: UGUUGUAAUAUUGCACCUCTT
Syndecan-1-siRNA-1	Sense: GCGACGAGGAGGAGUACUA
	Antisense: UAGUACUCCUCCUCGUCGC
Syndecan-1-siRNA-2	Sense: GCCUGUUCUACGAGAAGAA
	Antisense: UUCUUCUCGUAGAACAGGC
Syndecan-1-siRNA-3	Sense: UGUGUUUUCAAACUGAUUGAG
	Antisense: CAAUCAGUUUGAAAACACAGG
siNC	Sense: GCUCUUCCUUACUUCUUAA
	Antisense: UUAAGAAGUAAGGAAGAGC

### Confocal microscopy

PK-15 cells were seeded into confocal culture dishes and cultured until reaching 40%–50% confluency. Also, the cells were co-transfected with the CD81-Flag and Syndecan-1-Myc plasmids. After 24 h of transfection, the liquid in the culture dishes was discarded, and the cells were washed three times with sterile PBS for 5 min each time. Subsequently, the cells were fixed with 4% paraformaldehyde at room temperature for 30 min, followed by removal of paraformaldehyde and treatment with 0.1% Triton X-100 (Biosharp, BS084) for cell permeabilization at room temperature for 10 min. The cells were then washed three times with sterile PBS for 5 min each time. Next, the cells were blocked with PBST containing 3% BSA (Bovine serum albumin) at room temperature for 1 h, followed by overnight incubation with the corresponding primary antibodies at 4°C. After washing the cells three times with sterile PBS, they were incubated with CoraLite488-conjugated Goat Anti-Mouse IgG (Proteintech, SA00013-1) and CoraLite594-conjugated Goat Anti-Rabbit IgG (Proteintech, SA00013-4) at room temperature in the dark for 1.5 h. Subsequently, the cells were washed three times with sterile PBS and incubated with DAPI staining solution at room temperature in the dark for 5 min. Finally, the culture dishes were observed under a confocal microscope.

### Blocking experiment with anti-CD81 extracellular domain antibody

PK-15 cells were seeded in 24-well plates. When the cell density reached 70%–80% confluency, the medium in the wells was removed and the cells were washed three times with sterile PBS. Different final concentrations of anti-CD81-LEL antibody or isotype control antibody diluted in DMEM were added. After 1 h of incubation at 37°C in a cell culture incubator, the liquid in the wells was removed, and the cells were washed three times with DMEM before being infected with 0.1 MOI PCV2. Following a 1-h incubation at 37°C, the medium was replaced with maintenance medium. The presence of antibodies in the medium was maintained throughout the experiment. Protein samples were harvested 36 h post-infection for Western blot analysis.

### Experiment on inhibition of virus infection by recombinant CD81-LEL protein

The purified recombinant proteins CD81 LEL (aa 114-201) and unrelated PRV UL49 expressed from the pET28a vector in *Escherichia coli* in the form of inclusion bodies contained His tag and thrombin at N terminal and His tag in C terminal, and named as CD81 LEL-His and UL49-His, respectively. They were diluted to different concentrations, then mixed with 0.1 MOI PCV2 and incubated at 37°C for 1 h. PK-15 cells were seeded onto a 24-well plate. Upon reaching 70%–80% confluency, the medium in the wells was removed. The cells were washed three times with sterile PBS, followed by inoculation with the aforementioned protein-virus mixture. After 1 h of incubation at 37°C in a cell culture incubator, the medium was replaced with maintenance medium. Protein samples were harvested 36 h post-viral infection for Western blot analysis.

### Virus adsorption assay

The CD81 knockdown PK-15 cell line and wild-type PK-15 cells were separately seeded onto 12-well plates. Upon reaching confluency, cells were pre-chilled at 4°C for 30 min. Subsequently, the culture medium was discarded, and cells were washed three times with pre-chilled sterile PBS. Cells were then inoculated with 5 MOI PCV2 and incubated at 4°C for 1 h. After 1 h, the viral inoculum was removed, and cells were washed three times with pre-chilled sterile PBS to remove unbound virus particles. Total DNA samples were extracted, with cellular mtDNA (Mitochondrial DNA) as an internal reference, for qPCR analysis. The relative quantities of virus particles bound to the surface of knocked-down cells versus wild-type cells were compared.

### Virus internalization assay

CD81 knockdown PK-15 cell line and wild-type PK-15 cells were separately seeded into 12-well plates. Upon reaching confluence, cells were pre-chilled at 4°C for 30 min. Subsequently, the culture medium was discarded, and cells were washed three times with pre-chilled sterile PBS. Cells were then inoculated with 5 MOI PCV2 and incubated at 4°C for 1 h. After 1 h, cells were thoroughly washed with pre-chilled sterile PBS to remove unbound virus particles. Cells were then transferred to a 37°C cell culture incubator and incubated for an additional 2 h. After 2 h, the liquid in the wells was discarded, and cells were washed three times with pre-chilled citric acid buffer (pH = 3). Subsequently, cells were treated with 0.25% trypsin for 5 min to remove virus particles that did not internalize into the cells. Cells were collected, and total DNA samples were extracted, with cellular mtDNA (Mitochondrial DNA) as an internal reference, for qPCR analysis. The relative quantities of virus particles internalized into the knockdown cells versus wild-type cells were compared.

### Construction of CD81 and RhoA knockdown cell lines

Using the shRNA online design tool (https://rnaidesigner.thermofisher.com), shRNAs targeting porcine CD81 and RhoA genes were designed and the sequences were submitted to Nanjing GenScript Company for synthesis. The shRNA primer sequences are listed in Table 4. The synthesized shRNA fragments were cloned into the pLKO.1 vector and co-transfected with psPAX2 and pMD2.G plasmids into HEK-293T cells for lentivirus packaging. After 48 h of transfection, lentivirus was collected from the supernatant and used to infect PK-15 cells. Following a 16-h infection period, the culture medium was replaced with fresh medium and cells were further cultured for 48 h. Subsequently, the medium was replaced with maintenance medium containing 10 µg/mL puromycin to select cells stably expressing the target gene shRNA. The selected cells were subjected to Western blot analysis for identification, and named as shCD81 and shRhoA, followed by expansion in culture for subsequent experiments.

**TABLE 4:** shRNA sequences

Primer	Sequence (5′→3′)
CD81-shRNA-1	Sense: CCGGGCATCAAGTACCTGCTCTTCGCTCGAGCGAAGAGCAGGTACTTGATGCTTTTTG
	Antisense: AATTCAAAAAGCATCAAGTACCTGCTCTTCGCTCGAGCGAAGAGCAGGTACTTGATGC
CD81-shRNA-2	Sense: CCGGGCAACATCATCAGCAACTTGACTCGAGTCAAGTTGCTGATGATGTTGCTTTTTG
	Antisense: AATTCAAAAAGCAACATCATCAGCAACTTGACTCGAGTCAAGTTGCTGATGATGTTGC
CD81-shRNA-3	Sense: CCGGGGGTGCTGTGATGATGTTTGTCTCGAGACAAACATCATCACAGCACCCTTTTTG
	Antisense: AATTCAAAAAGGGTGCTGTGATGATGTTTGTCTCGAGACAAACATCATCACAGCACCC
RhoA-shRNA-1	Sense: CCGGGCCTGTGGCAAGACTTGTTTGCTCGAGCAAACAAGTCTTGCCACAGGCTTTTTG
	Antisense: AATTCAAAAAGCCTGTGGCAAGACTTGTTTGCTCGAGCAAACAAGTCTTGCCACAGGC
RhoA-shRNA-2	Sense: CCGGGCAAGACTTGTTTGCTCATTGCTCGAGCAATGAGCAAACAAGTCTTGCTTTTTG
	Antisense: AATTCAAAAAGCAAGACTTGTTTGCTCATTGCTCGAGCAATGAGCAAACAAGTCTTGC
RhoA-shRNA-3	Sense: CCGGGGCTGCCATCAGGAAGAAACTCTCGAGAGTTTCTTCCTGATGGCAGCCTTTTTG
	Antisense: AATTCAAAAAGGCTGCCATCAGGAAGAAACTCTCGAGAGTTTCTTCCTGATGGCAGCC

### Statistical analysis

All data were analyzed using GraphPad Prism 9.0 with one-way analysis of variance (ANOVA), two-way ANOVA, or Student’s *t*-test. Data are presented as mean ± SD from three independent replicate experiments. *P*-values are denoted as follows: *, *P* < 0.05; **, *P* < 0.01; ***, *P* < 0.001; ns indicates non-significant differences between groups.

## Data Availability

The data supporting the findings of this study are available within the article and its supplemental material.

## SUPPLEMENTAL MATERIAL

Supplemental legend jvi.01408-24-s0001.docx Legend for supplemental table.

Supplemental table jvi.01408-24-s0002.xlsx Mass spectrometry data.

ASM does not own the copyrights to Supplemental Material that may be linked to, or accessed through, an article. The authors have granted ASM a non-exclusive, world-wide license to publish the Supplemental Material files. Please contact the corresponding author directly for reuse.
