# Supplementary material for: Tetraspanin CD81 serves as a functional entry factor for porcine circovirus type 2 infection
Source: J Virol. 2024 Dec 31;99(2):e01408-24. doi: 10.1128/jvi.01408-24 (PMC11853000; doi:10.1128/jvi.01408-24)
Supplement: Supplemental legend — Legend for supplemental table. [file jvi.01408-24-s0001.docx]

**Supplemental Table Legend**

The protein information from the mass spectrometry analysis of the silver-stained gel strip in Fig. 1A.
